# Comparing oral health systems for children in six European countries to identify lessons learned for universal oral health coverage: A study protocol.

**DOI:** 10.12688/hrbopenres.13458.1

**Published:** 2022-01-17

**Authors:** Úna McAuliffe, Noel Woods, Shauna Barrett, Jodi Cronin, Helen Whelton, Máiréad Harding, Kenneth Eaton, Sara Burke

**Affiliations:** 1 Oral Health Services Research Centre, University College Cork, Cork, Co Cork, T12E8YV, Ireland; 2 School of Public Health, University College Cork, Cork, Co Cork, T12K8AF, Ireland; 3 Centre for Policy Studies, Cork University Business School, Cork, Co Cork, T12EP08, Ireland; 4 Cork University Hospital Library, Cork University Hospital, Wilton, Cork, T12 DC4A, Ireland; 5 College of Medicine and Health, University College Cork, Cork, Co. Cork, T12EDK0, Ireland; 6 Cork University Dental School and Hospital, Wilton, Cork, T12EYV, Ireland; 7 Eastman Dental Institute, University College London, 21 University Street, London, WC16DE, UK; 8 University of Kent, Chatham Maritime, ME4 4AG, UK; 9 Centre for Health Policy and Management, School of Medicine, Trinity College Dublin, The University of Dublin, College Green, Dublin 2, D02 PN40, Ireland

**Keywords:** Oral health systems, universal health coverage, childhood dental caries, oral health policy, health system financing, protocol, reform.

## Abstract

**Background: **Oral diseases have the highest global prevalence rate among all diseases, with dental caries being one of the most common conditions in childhood. A low political priority coupled with a failure to incorporate oral health within broader health systems has contributed to its neglect in previous decades. In response, calls are emerging for the inclusion of oral health within the universal healthcare domain (UHC). This protocol outlines the methodology for a cross-country comparative analysis of publicly funded oral health systems for children across six European countries, reporting on oral health status in line with the indicators for UHC.

**Methods: **This study will follow Yin’s multiple case study approach and employ two strands of data collection, analysis, and triangulation: a systematic documentary analysis and semi-structured interviews with elite participants local to each country. The countries chosen for comparison and providing a representative sample of European dental systems are Denmark, Hungary, the Republic of Ireland, Germany, Scotland, and Spain. A systematic search of five electronic databases and four additional electronic resources will be undertaken, in addition to grey literature and other publicly available sources, with the outcomes verified and further informed by local experts. The WHO Universal Health Coverage Cube will be used to guide data collection and analysis.

**Conclusions: **This research will provide policy makers with an in-depth analysis and comparison of publicly funded oral health systems for children in Europe, including consideration of effective preventive strategies, oral health system reform, and indicators of universal oral health coverage. It is anticipated that the outcomes may help in positioning oral health on governmental health agendas and support its integration into wider health systems’ reform in an accessible and affordable manner.

## Introduction

Oral diseases, despite being largely preventable, are among the most prevalent non-communicable diseases globally ( World Health Organization, 2020). People are impacted from childhood to adolescence, adulthood and into later life with little improvement of the situation over the past two decades ( World Health Organization, 2020). Furthermore, the burden of oral disease is hallmarked by significant inequality, disproportionately affecting marginalised populations and those in low socio-economic groups ( Peres *et al*., 2019).

Dental caries (tooth decay) is an especially serious public health problem for children, affecting 60–90% of schoolchildren worldwide ( Petersen & Lennon, 2004; Slade *et al.,* 2018). In addition to causing pain and infection, caries significantly impact children’s social and psychological wellbeing, along with their quality of life ( BaniHani *et al.,* 2018; Peres *et al.,* 2019). It is associated with reduced school attendance, impaired speech development and can result in lower body weight and height ( Jackson *et al.,* 2011; Sheiham, 2006).

Dental caries not only impacts the affected child, but also the broader family, resulting in lost workdays with the added financial burden that may be associated with accessing dental services ( Casamassimo *et al.,* 2009). Furthermore, the management of dental caries in younger children may require hospitalisation for treatment under general anaesthesia with associated health, financial and health system implications ( McAuliffe *et al.,* 2017; Peres *et al.,* 2019). This is a problem of global concern, with the management of oral diseases and extraction of carious (decayed) teeth previously cited as the number one reason for hospital admission of young children in Australia and England ( Chrisopoulos, 2016; Levine, 2021).

Important risk factors for disease development in children have been identified, most notably high-sugar dietary habits ( Watt *et al.*, 2019) with parental behaviour being a ‘significant predictor’ ( Östberg *et al*., 2017), while fluoride exposure is an important protective factor ( Petersen & Lennon, 2004). Early availability and access to preventive oral healthcare has been proposed as fundamental to improving children’s oral health, reducing costs, and establishing life-long practices ( Irish Oral Health Services Guideline Initiative, 2012; World Health Organization, 2020).

In May 2021, the World Health Assembly adopted a historic resolution on oral health, strongly advocating for its inclusion under the Universal Healthcare (UHC) agenda ( World Health Organization, 2021a). The WHO defines UHC as ensuring ‘all people have access to services and do not suffer financial hardship for paying for them’ ( World Health Organization, 2021b). Evidence has suggested that UHC may be a global risk indicator for early childhood caries (ECC), i.e., dental caries diagnosed in children under age six ( Anil & Anand, 2017) with a lower prevalence of ECC seen in countries with good UHC ( El Tantawi *et al.,* 2018). However, not all countries with UHC include entitlements to oral healthcare, particularly preventative oral healthcare, which is often considered a non-essential health service ( Wang *et al.,* 2020).

Historically, oral healthcare was relegated to the realm of personal responsibility ( Wang *et al.,* 2020) and currently remains a low priority across governments globally. This has led to a lack of political commitment, coverage, and a failure to resource disease prevention ( World Health Organization, 2020). Responding to the ‘global state of crisis’ in oral health requires a shift in focus from the traditionally curative models of care to a more inclusive approach, where oral health services are fully integrated in universal health systems worldwide ( Glick *et al.,* 2021; Watt *et al.,* 2019).

However, dental caries is a complex, multifactorial disease influenced by biological factors and the social determinants of health ( Meyer & Enax, 2018; Watt *et al.,* 2019). There have been conflicting results demonstrating the influence of access to oral healthcare on the prevalence of dental caries in very young children, with a reduction in disease severity evident in Nordic countries with universal oral health cover ( Virtanen *et al.,* 2007), yet a high disease prevalence remaining, or no impact seen, in countries with free access like Peru and Brazil ( Azañedo *et al.,* 2017; Pucca Jr *et al.,* 2015). Recent research by Folayan *et al*. (2021), found that higher governmental health expenditure may be associated with a reduction in ECC and called for further research across individual countries to assess the impact of UHC on disease prevalence, and how UHC could be optimised to reduce the risk of dental caries.

In Europe, healthcare systems are publicly financed via general taxation or compulsory social health insurance and complemented by private contributions from either voluntary insurance and/or direct out of pocket payments (OPP) by individuals ( Sinclair *et al.,* 2019). Based on the method of administration and financing, six oral healthcare systems have been described, namely the Nordic, Bismarkian, Beveridgian, Southern European, Eastern European and Hybrid models ( Widström & Eaton, 2004). However, the extent of public financing, universal coverage and the density and distribution of dentists working at the primary healthcare level varies within each system ( Glick *et al.,* 2021; Patel, 2012; The Platform for Better Oral Health in Europe, 2015).

In terms of publicly funded oral healthcare for children, the child population covered and the extent of cover also varies within and across systems ( Table 1). For example, Denmark and Germany, representative of the Nordic and Bismarkian systems respectively, provide free basic oral healthcare services to pre-school and school children where even the youngest children are included in the system ( Widström & Eaton, 2004). The Danish system is largely tax-financed, while Germany has a statutory health insurance system split into public social insurance (SHI) and private health insurance ( Ziller *et al.,* 2015). In the Beveridgian system, unique to the United Kingdom, the entire population is eligible to obtain treatment through the tax-financed National Health Service (NHS). In 2006, the NHS payment system changed in England, Wales, and Northern Ireland. However, it remained the same in Scotland, and oral healthcare treatment including orthodontics is free of charge to all patients under the age of 18 years as at time of writing ( Sinclair *et al.,* 2019).

**Table 1.** An overview of oral health systems coverage for children in six European countries.

Country	Main source of general healthcare financing, model and percentage of current expenditure financed in 2019. TF = Tax financed, SHI= Social health insurance.	Oral healthcare model	Children’s oral health cover
**Denmark**	TF (83.3%)	Nordic	Universal
**Hungary**	SHI (59.8%)	Eastern European	Universal
**Ireland**	TF (74%)	Hybrid	At specific ages with restrictions
**Germany**	SHI (78.1%)	Bismarkian	Universal
**UK**	TF (78.5%) *	Beveridgian	Universal
**Spain**	TF (66.6%)	Southern European	At specific ages with restrictions

*The figure for the UK is for England, Scotland and Wales. The table saying the focus of this analysis is Scotland. *Source: Sinclair *et al.* (2019) and Eurostat (2021)*. * Office for National Statistics, 2021, UK Health Accounts 2019, UK https://www.ons.gov.uk/peoplepopulationandcommunity/healthandsocialcare/healthcaresystem/bulletins/ukhealthaccounts/2019#total-current-healthcare-expenditure-in-the-uk

In contrast, the Spanish health system, an example of a South European system, is primarily tax-financed and organised nationally and regionally, with health competencies transferred to the 17 Autonomous Communities (ACs) ( Bravo *et al.,* 2015). The level of oral health coverage for Spanish children strongly depends on regional location, with some oral healthcare available to children aged from seven to 15 years of age in certain areas yet little coverage in other regions ( Sinclair *et al.,* 2019). In most Eastern European countries, the provision of care has shifted from largely free public provision to the majority of care being delivered in the private sector ( Widström & Eaton, 2004). Hungary, an example of an Eastern European system, is financed by contributions from employed persons and employers with children receiving free oral healthcare until the age of 18 ( Kivovics & Csado, 2010).

In the Republic of Ireland (Ireland), representative of a hybrid system, children are targeted for comprehensive care at specific age groups, with restricted access other than emergency treatment for all other cohorts ( Department of Health, 2019). Ireland’s general health system is complex and predominantly funded through general taxation. However, more than half of the Irish population purchase private health insurance, predominantly to facilitate quicker access to hospital care and private healthcare ( Johnston *et al.,* 2020). In contrast to the general health system, two thirds of all dental expenditure in Ireland is privately financed with some, but little, publicly funded coverage for oral healthcare ( Henry *et al.,* 2021; Johnston *et al.,* 2020). In any system when costs are high, children of limited means, who are also at increased risk, may not be able to afford care ( Watt *et al.,* 2019). This can result in inequalities in access to services and ultimately poorer oral health ( Wang *et al.,* 2020). 

In addition to the range of differences between systems, including funding, there is a paucity of information on how the system is organised; for example, how services are delivered, the healthcare professionals responsible for providing care and access to services ( Allin *et al.,* 2020; Eaton *et al.,* 2019; Glick *et al.,* 2021). An in-depth understanding and comparison of how different oral care systems operate and their position within the UHC domain could provide a greater knowledge for healthcare planning and policy decision making ( Eaton *et al.,* 2019; Folayan *et al.,* 2021).

### Aim of this research

The primary aim of this research is to compare publicly funded oral health coverage for children under the age of eighteen years across six European countries, each of which is representative of a model for the provision of oral healthcare, and to report on oral health status in line with the indicators for universal oral healthcare.

This protocol outlines the methodology for the collection, management, and analysis of data from a systematic documentary analysis and semi-structured interviews with local experts, pertaining to Denmark, Hungary, Ireland, Germany, Scotland, and Spain, to meet the primary study aim.

### Objectives of this research

1.   To describe and compare models of publicly funded oral health systems for children across the chosen six European countries including the financing arrangements associated with each system.

2.   To collect and compare available and relevant indicators of universal coverage for oral health in the chosen countries.

3.   To identify those national oral healthcare systems with effective oral health prevention and promotion programmes for children, as evidenced by low d _3_mft/D _3_MFT or ICDAS 0-3 and the percentage (%) with no obvious dental caries at age 12.

4.   To learn from successful examples of oral health systems for children internationally, in terms of outcomes and costs, that can be applied to other countries.

## Protocol

### Study design

This is a cross-national comparative analysis of publicly funded oral health systems for children under the age of eighteen across six European countries. The study will follow Yin’s multiple-case study approach ( Yin, 2014). To strengthen study validity, two strands of data collection, analysis and triangulation will be undertaken:

1. A systematic documentary analysis.
2. Semi-structured interviews with ‘elite’ participants from each country ( Van Audenhove & Donders, 2019).

### Ethical approval

Ethical approval for the study was obtained from the Clinical Research Ethics Committee of the Cork Teaching Hospitals at University College Cork (Reference number: ECM 4 (J)12/11/2019). Written informed consent to participate will be sought from each participant prior to interview.

### Country selection

Countries proposed for comparison are:

1. Nordic system: Denmark
2. Eastern European system: Hungary
3. Hybrid system: Ireland
4. Bismarkian system: Germany
5. Beveridgian system: Scotland ^1^
6. Southern European system: Spain

***Criteria for country selection***

The criteria for country selection are those:

1. Which are representative of the variation of health systems throughout Europe (i.e., social health insurance vs. tax-financed, multi- vs single-payer, centralised versus decentralized) and the different models of dental systems in Europe ( Sinclair *et al.,* 2019).
2. Demonstrate effective oral health prevention and promotion strategies evidenced by low d _3_mft/D _3_MFT and percentage with no obvious caries at age 12 or ICDAS 0-3.
3. With a history of, or planned oral health system reform.

***Indices for assessing dental caries.*** The Decayed, Missing and Filled Teeth (DMFT) Index has been used for almost 80 years and remains the most commonly used epidemiological index for assessing dental caries ( Broadbent & Thomson, 2005). Obvious decay is when the disease appears to have penetrated dentine and is described as decay at the ‘d _3_ level’ in deciduous teeth, the ‘D _3_ level’ in permanent teeth, and includes pulpal decay. This definition is in accordance with international epidemiological conventions, thus facilitating optimum comparison. It is recommended that countries conduct periodic national oral health surveys, with the 12-year-old age group considered particularly important as a target group for assessing the level of dental caries severity among children with permanent teeth ( World Health Organization, 2013).

The International Caries Detection and Assessment System (ICDAS) was developed to provide a standardized caries detection and diagnosis system across a range of different environments ( Pitts & Stamm, 2004). The ICDAS measures changes and potential lesion depth based on surface characteristics. The ICDAS scores range from zero to six for coronal caries, with zero representing a sound tooth and six an extensive distinct cavity with visible dentin ( Shivakumar *et al.,* 2009).

There are limitations associated with the DMFT, particularly the range of diagnostic criteria, sampling techniques and methods used to train and calibrate examiners across different countries. Nationally reported DMFT varies across countries, with some reports stemming from data generated almost 20 years apart ( Patel *et al.*, 2016). This will be recognised in the findings of this study by highlighting the year of reporting to facilitate appropriate interpretation of each country’s D _3_MFT.

### Participants

In-depth interviews will be undertaken with individuals selected as representatives of, and with expert knowledge local to, each country. Participants will be identified via (i) the outcomes of the documentary analysis, (ii) using purposive sampling based on the recommendations of participants and (iii) the researchers’ understanding of individuals with expert knowledge of the oral health system in question. It is anticipated that two experts from each country, i.e., a total of twelve experts *(n=12)*, will participate.

### Data collection

**Figure 1.**
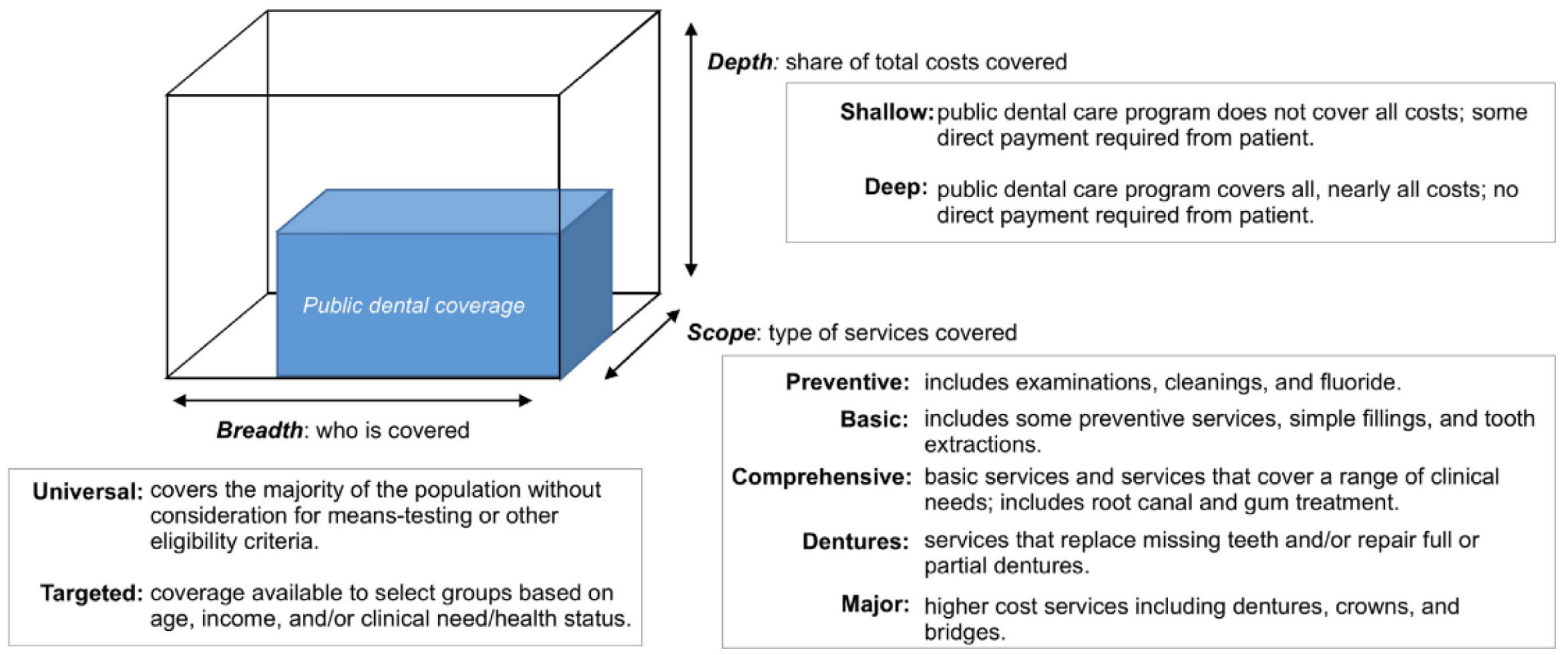
Dimensions of coverage for public oral healthcare models based on the World Health Organization Coverage cube framework. Adapted from Allin *et al*., 2020.

***Conceptual framework.*** To describe and compare publicly funded oral health systems for children under age 18 across the six chosen countries, each model will be described according to the three core features of the WHO Coverage Cube Framework ( World Health Organization, 2010): (i) breadth, i.e., child populations (0–18 years) eligible for publicly funded oral health programs; (ii) depth, i.e., the share of the total costs that are borne by the government/public payer; and (iii) scope, i.e., the range of services covered under publicly funded oral healthcare programs ( Figure 1).

### Documentary analysis

A documentary analysis is a systematic procedure for reviewing documents, requiring data be examined and interpreted to elicit meaning and gain empirical understanding of a given topic ( Bowen, 2009). This systematic documentary analysis will aim to examine the relevant reports, policies, service documents, academic publications and other literature developed at local, regional, and national level for each country.

***Information sources***

The relevant documents will be identified in the following ways:

1. A literature search of the following databases: Medline (EBSCO), CINAHL (EBSCO), EMBASE, ProQuest and the Cochrane Library. The TRIP database will be searched for grey literature along with medRxiv (for health sciences preprints), while Google Scholar and Web of Science will also be utilised.
2. Manual reference checks of identified documents and studies.
3. Publicly available resources and documents will be searched to identify existing reviews, oral health policies and position papers regarding oral health coverage by national and international stakeholders e.g., governmental health departments of each country and international organisations including the World Health Organization, FDI World Dental Federation and the Council for European Chief Dental Officers.

***Search strategy.*** The search strategy for this research will be developed with the assistance of a specialist medical librarian (SKB) based at Cork University Hospital, Cork, Ireland, in collaboration with the lead author, an academic dental researcher (UM). The search strategy will entail using a combination of keywords and subject headings, incorporating MeSH (PubMed/Medline), CINAHL Headings (CINAHL) and EMTREE headings (EMBASE). Search results will be filtered for items published in English since 2001 and will then be screened by title and abstract. If key documents are only available in the country’s native language, these will be translated with verification of their critical elements sought from local experts. The search strategy presented in extended data ( McAuliffe, 2021) is based on Medline and will be tested and adapted for all other databases and resources. An overview of the MeSH terms is presented in Table 2.

**Table 2.** Specific MeSH terms employed.

Area of interest	MeSH terms
**“Health** ** Indicators”**	(MH “Quality Indicators, Health Care+”)
**“Population** ** under age 18”**	(MH “Child+”) OR (MH “Child, Preschool”) OR (MH “Infant+”) OR (MH “Infant, Newborn+”) OR (MH “Adolescent”) OR (MH “Young Adult”)
**“Universal** ** Health Care”**	(MH “Universal Health Insurance”) OR (MH “Universal Health Care”) OR (MH “Delivery of Health Care+”) OR (MH “National Health Programs+”) OR (MH “Insurance, Dental”) OR (MH “Global Health”)
**“Healthcare ** **Costs”**	(MH “Socioeconomic Factors+”) OR (MH “Health Expenditures+”) OR (MH “Cost-Benefit Analysis”) OR (MH “Health Care Costs+”)
**“Oral Health”**	(MH “Dental Caries+”) OR (MH “DMF Index”) OR (MH “Dental Care+”) OR (MH “Dental Care for Children”) OR (MH “Preventive Dentistry+”) OR (MH “School Dentistry”) OR (MH “State Dentistry”) OR (MH “Pediatric Dentistry”) OR (MH “Tooth Diseases+”) OR (MH “Mouth Diseases+”) OR (MH “Tooth, Deciduous+”) OR (MH “Fluoridation”)
**“Country”**	(MH “Ireland”) OR (MH “Spain”) OR (MH “Germany+”) OR (MH “Denmark+”) OR (MH “Hungary”) OR (MH “Scotland+”) OR (MH “Europe+”) OR (MH “Europe, Eastern+”) OR (MH “European Union”)

***Inclusion criteria***

**Types of study to be included**

No limitations will be placed on the study or document included if the study design and document address the research aim. Government documents such as national oral health policies along with clinical guidelines academic literature, oral health service reports, and other grey literature, in addition to international recommendations pertaining to evidence for improved oral health, will be eligible for inclusion.

**Population, concept, and context**

This research seeks to compare oral health systems for children across countries. To ensure most systems are eligible for consideration, an age range up to 18 years is proposed. It is beyond the scope of this research to consider oral health service reviews for special needs and other vulnerable populations. Policies, guidance, and other literature that pertain to public oral healthcare systems, strategies for oral health promotion and prevention, universal oral health coverage, dental system reform and improved oral health status for children ≤ 18 years will be included. All included studies/documents must include two or more of the following outcomes: (1) publicly funded dental systems seeking to, or who have successfully, implemented universal oral health coverage in the last twenty years, (2) reduced oral health inequalities, (3) improved oral health system accessibility (4) reduced oral health system costs (5) caries rates as determined by d _3_mft/D _3_MFT and percentage with no obvious caries at 12 years, or (6) the International Caries Detection and Assessment System (ICDAS) and other indicators of oral health status including periodontal diseases and oral health-related quality of life measures (OHRQoL). Please see Table 3 for full inclusion criteria.

**Table 3.** Documentary analysis related inclusion criteria.

Criterion	Inclusion
**Population**	Community dwelling children aged less than 18 years of age.
**Document/study type**	No limitations on document type provided research aim is addressed. Grey literature including national oral health policies, health service reports and recommendations will also be included
**Setting**	Publicly funded oral healthcare systems including primary and secondary care. *Excluded: residential oral healthcare facilities*
**Outcome(s)**	Included documents must have two or more: 1. Publicly funded oral health systems successfully implemented UHC 2. Reduced oral health inequalities 3. Reduced oral health system costs 4. Improved oral health system accessibility 5. Dental caries rates via dmft/D3MFT and % with no obvious caries at age 12 or ICDAS (0–3) 6. Other indicators of oral health status including periodontal diseases/Oral Health Related Quality of Life measures
**Time period**	2001–2021
**Language**	English

***Study selection and data extraction***

**Download of title and abstract records**

Titles and abstracts identified will be downloaded in file formats usable with our chosen reference management software (Zotero) with any additional information stored in Microsoft Excel. They will then be uploaded to Zotero and Rayaan, an online application supporting systematic review screening efficiency ( Ouzzani *et al.*, 2016), with any duplicate entries removed.

**Reviewer calibration**

A data charting form will be developed, guided by the inclusion criteria. The data selection process and form will be pilot-tested in 10% of randomly selected and included articles to assess reviewer calibration. Any discrepancies between reviewers will be discussed with amendments made to the data extraction form and further calibration as required to ensure near perfect (>80%) agreement.

**Title and abstract reviews**

Title and abstract reviewing will be performed by one reviewer (UM) and verified by another (JC). The verification process will entail an assessment of the original screening of the title and abstract to confirm whether the second reviewer agrees with the decision made. Any discrepancies will be discussed with a third researcher engaged (SB or KE) if a consensus fails to be reached. Titles and abstracts that fail to meet the eligibility criteria will be excluded from the next stage of assessment, while those that conform to the eligibility criteria will be included for full-text review.

**Full-text review**

Full-text material will be sourced using the resources provided by University College Cork. If the full text cannot be accessed, the team will investigate if they can be retrieved via any other library to which the broad team has access, via international experts local to each country or by contacting the relevant authors.

**Data extraction**

A data extraction form will be developed, guided by the WHO Coverage Cube, based on previous work by Allin *et al*., 2020, and further refined for this research. Full-text reviews will be completed by one reviewer (UM) with verification by another (JC). Any discrepancies will be resolved by discussion, and failure to reach consensus will require consultation with a third member of the research team (SB or KE). The Preferred Reporting Items for Systematic Reviews and Meta-Analyses (PRISMA-S) will be applied in illustrating this approach ( Rethlefsen *et al.*, 2021).

***Risk of bias (quality) assessment.*** Risk of bias will be assessed by one researcher and verified by another with any discrepancies resolved in discussion with a third member of the research team (SB or KE). Each study will be assessed by a tool appropriate for its study design, particularly using the Critical Appraisal Skills Programme (CASP) checklists pertaining to the individual study type. However, considering the nature of the research question, not all items are applicable to every type of included document in this analysis and quality ratings will be determined in this instance.

### Semi-structured interviews with elite participants

Following the outcomes of the documentary analysis and guided by the WHO Coverage Cube, a semi-structured interview guide will be developed for use with all participants ( World Health Organization, 2010). This will be piloted and further reviewed in structured meetings with the research team. The full list of participants will be regularly updated throughout the research process. In view of the wide geographic spread of participants, it is expected that all interviews will take place via tele-conferencing tools.

### Strategy for data analysis

This research will follow Yin’s multiple-case study methodology in employing two strands of data collection and analysis ( Yin, 2014).

Document analysis requires superficial examination, thorough examination, and interpretation of documents combining content analysis and thematic analysis ( Bowen, 2009). This analysis will follow a directed content analysis approach guided by the WHO framework and employ a deductive category application ( Graneheim & Lundman, 2004). This research will follow the checklist to improve trustworthiness in content analysis developed by Elo *et al*. (2014).

Key initial concepts will be identified as coding categories using the WHO framework as guidance before operational definitions for each category are defined. Interviews will be transcribed verbatim and coded. All data will be managed and coded using qualitative data analysis software (NVivo 12 qualitative data analysis software).

The data from the documentary analysis will be analysed together with the data from interviews to ensure themes are triangulated across both datasets. A dedicated case study will then be drafted for each country individually before cross case analysis will be conducted to identify patterns of similarities and differences across the cases. All sub-themes and main themes will be tabulated and discussed among the research team, with a particular emphasis on the possible implications for policymakers.

The potential limitations of documentary analyses include: an absence of detail, low retrievability or biased selectivity ( Yin, 1994). Additionally, interview data may be subjective and ambiguous ( Pannucci & Wilkins, 2010). It is expected the data triangulation employed in this research will guard against the possible limitations of trustworthiness, researcher bias and respondent bias. Additional techniques including a documented systematic search, a ‘thick’ description of outcomes and an audit trail will further serve to ensure verification.

### Dissemination

The findings will be presented to policymakers and governmental health departments in each country, in addition to professional networking conferences both nationally and internationally, such as the European Association of Dental Public Health congress.

### Study status

The search strategy is under development with the support of a specialist medical librarian (SKB) at Cork University Hospital with initial searches of the MEDLINE database underway.

## Conclusion

The challenges to meeting oral health needs of populations, and particularly vulnerable groups, is now receiving global attention, with policy makers being urged to appropriately position oral health within the emerging universal healthcare (UHC) agenda ( Glick *et al.,* 2021; Watt *et al.,* 2019; World Health Organization, 2021a). However, there is conflicting support for the impact of improved access to oral health systems and the prevalence of dental caries in children ( Pucca Jr *et al.,* 2015; Virtanen *et al.,* 2007). Recent evidence has confirmed that higher governmental health expenditure may be associated with lower prevalence of the disease among very young children; however, calls have been made for country-specific studies to determine how UHC may reduce the risk of dental caries ( Folayan *et al.,* 2021). This proposed research aims to compare publicly funded oral health systems for children throughout Europe and to report on oral health status in line with the indicators for UHC. By providing this evidence for oral health advocates internationally, this research may support the positioning of oral health on governmental agendas and ensure that essential oral health systems are integrated into broader healthcare in an accessible and affordable manner.

## Data availability

### Underlying data

No data are associated with this article.

### Extended data

Open Science Framework: Comparing oral health systems for children in six European countries to identify lessons learned for universal oral health coverage: A study protocol: Extended data.,

https://osf.io/347vw

This project contains the following extended data:

- Medline (EBSCO) Search strategy.docx (The mix of key words and mesh terms that will be used to search Medline and that will be transferred to other databases for searchers).

Data are available under the terms of the Creative Commons Zero "No rights reserved" data waiver (CC0 1.0 Public domain dedication).
