# Supplementary material for: Comparing oral health systems for children in six European countries to identify lessons learned for universal oral health coverage: A study protocol
Source: HRB Open Res. 2022 Jan 17;5:5. [Version 1] doi: 10.12688/hrbopenres.13458.1 (PMC10520513; doi:10.12688/hrbopenres.13458.1)
Supplement: Peer review history

# Reviewer report: Reviewer response for version 1

**DOI:** 10.21956/hrbopenres.14666.r36052

**Published:** 2023-09-25

**Authors:** Xiaojuan Zeng

**Affiliations:** 1 Dental pubic Health, College & Hospital of Stomatology, Guangxi Medical University, Guangxi, China

This study will follow Yin’s multiple case study approach and employ two strands of data collection, analysis, and triangulation with elite participants local to each country. This study proposal is interesting and good. The background was sufficient writing. The study clearly described the aims and objectives. The study design was suitable for the study questions. The methods were good enough to guide other to do similar study. In addition, the datasets were clearly presented in a useable and accessible format.  

 This study may provide policy makers with effective preventive strategies, oral health system reform, and indicators of universal oral health coverage. It may help in positioning oral health on governmental health agendas and support its integration into wider health systems’ reform in an accessible and affordable manner. However, it will be great if more countries such as China can be involved in the future.

Is the study design appropriate for the research question?

Yes

Is the rationale for, and objectives of, the study clearly described?

Yes

Are sufficient details of the methods provided to allow replication by others?

Yes

Are the datasets clearly presented in a useable and accessible format?

Yes

Reviewer Expertise:

Oral epidemiology, Oral health promotion, Oral health related quality of life, Common risk factor on Oral disease and ChronicNon-communicable Diseases (NCD), Oral health policy, Diet and Oral health, Oral heath inequality

I confirm that I have read this submission and believe that I have an appropriate level of expertise to confirm that it is of an acceptable scientific standard.

# Reviewer report: Reviewer response for version 1

**DOI:** 10.21956/hrbopenres.14666.r36056

**Published:** 2023-09-25

**Authors:** Paulo Frazão

**Affiliations:** 1 University of São Paulo, São Paulo, Brazil

The authors present a study protocol that aims to compare publicly funded oral health coverage for children under the age of eighteen years across six European countries. The countries will be selected according to diversity of oral healthcare model that they deliver. Based on the management and financing strategy, six oral healthcare systems will be described, namely the Nordic, Bismarkian, Beveridgian, Southern European, Eastern European and Hybrid models. The rationale of the study is well formulated but some points deserve consideration. The literature shows that the magnitude of dental caries prevalence is strongly associated to sugar and fluoride exposures.

 Then it is important to clarify that the focus is untreated dental caries prevalence. This point is clearer in the Methods (use of ‘d3 level’ in deciduous teeth and the ‘D3 level’ in permanent teeth) than in the Introduction. This is particularly relevant because previous studies that sought to explore relationship between Universal Health Coverage (see that the manuscript reports incorrectly UHC as Universal Healthcare in the fifth paragraph, in the conclusion and in the abstract) and early childhood caries (ECC) have some major limitations. For example, the supporting studies (El Tantawi et al., 2018; Folayan et al. 2021) of the justification did not included indicators related to sugar and fluoride exposure. ECC’s definition requires the presence of one or more decayed (noncavitated or cavitated lesions), missing (due to caries), or filled tooth surfaces in any primary tooth. The conflicting results cited by the authors in the 7th paragraph can be related to methodological characteristics of the studies as the observed outcome and included variables. The presented study protocol will investigate dental caries determined by ‘d3 and D3 levels’ indicators.

 The study design will be based on multiple-case study approach in which a cross-national comparative analysis will be undertaken using documentary research and semi-structured interviews with local experts. Forward the authors stated that they will undertake in-depth interviews. So they will undertake semi-structured in-depth interviews. Each delivery model of oral healthcare will be described according WHO Coverage Cube Framework. Systematic documentary analysis will be performed examining scientific documents and technical reports. It was not justified why the authors will select local and regional reports.

 For the purpose of the protocol I think that national-level documents would be more appropriated. The procedures for searching and selecting of the documents were well described. The researchers will assess risk of bias using a tool appropriate for each study design. The presented protocol is well designed for producing evidence on publicly funded oral health systems for children throughout Europe and on oral health status in line with the indicators for Universal Health Coverage. It is important to highlight that UHC means Universal Health Coverage instead of Universal Health Care. The UHC strategy sponsored by WHO is not necessarily the same strategy implemented by some countries where the access to health services does not depend on the user's ability to pay. I agree with the authors that the current protocol may support the positioning of oral health on governmental agendas and ensure that essential oral health systems are integrated into broader healthcare in an accessible and affordable manner.

Is the study design appropriate for the research question?

Yes

Is the rationale for, and objectives of, the study clearly described?

Yes

Are sufficient details of the methods provided to allow replication by others?

Yes

Are the datasets clearly presented in a useable and accessible format?

Not applicable

Reviewer Expertise:

Oral health policy and epidemiology

I confirm that I have read this submission and believe that I have an appropriate level of expertise to confirm that it is of an acceptable scientific standard.

# Reviewer report: Reviewer response for version 1

**DOI:** 10.21956/hrbopenres.14666.r36059

**Published:** 2023-09-19

**Authors:** Kaung Myat Thwin

**Affiliations:** 1 Niigata University, Niigata, Japan

**Abstract:**

 Background: It would have been better if the types of oral health systems were mentioned (tax-financed and social health insurance).

 Methodology: Instead of stating that the study will follow Yin’s multiple case study approach, it would be better to state what information will be extracted from the literature (main source of general healthcare financing, the level of coverage (age), range of services), how it will be graded (risk of bias) or analyzed.

**Introduction:**

 The Introduction is quite long. It would have been better if the introduction of oral diseases and dental caries was brief. Since the title is about oral health systems, it would be more interesting to discuss why it was necessary to compare oral health systems for children in six European countries (research gap). Instead of discussing each country’s oral health systems, it would have been more organized to read which country utilizes tax financing and social health insurance as the main source of general healthcare financing, the level of coverage (age), range of services (included treatments), and so on.

**Aims**

1. Objectives: The statement “to learn from successful examples” limits the learning to only the success of implementing the oral health system. In my opinion, we can also learn even from not-successful examples. It is better to revise this sentence.
2. Please add the study’s hypothesis.

**Protocol:**

1. Study design: The statement “oral health systems for children under the age of eighteen” is vague. It is better to put the age range of the target population of this study.
2. What do “elite” participants mean? It would be easier to understand it if it was defined in item #2.
3. Indices for assessing dental caries: Please also mention why you will use two indices: DMFT and ICDAS.
4. In data collection, you mentioned that the children under age 18, while in Population, concept and context of Inclusion criteria, the children ≤18. Which one is correct? Please amend it.

**Inclusion criteria: **

1. What is the included literature? Does “No limitations on document types” mean you will include even study protocol or unpublished study?
2. Why do you limit 2001, is there any reason?
3. It would be better if you clarify whether you will include documents only in English or not since you described “If key documents are only available in the country’s native language, these will be translated with verification of their critical elements sought from local experts” in search strategy section.

**Data extraction**: It would be better if you provided what data will you extract from the articles.

**Risk of bias:** Since you include all types of articles, the CASP checklist cannot be performed in all included documents. In that case, what tools or checklist will you use?

 It would be better to state the research questions you want to answer in this review because study selection and data extraction will depend on it. The risk of bias assessment will be unclear without considering each research question.

**Conclusion:** Since there were four objectives mentioned in this study, it would have been better if each objective was addressed in the conclusion. Future study recommendations could also be mentioned (e.g., can be used for reform).

 Beside the above mentioned comments, I may suggest you to add the sections such as "Expected results", "Study period and study protocol".

 In addition, when you described the outcomes, it is better to specify which is primary outcome, then which are the secondary outcomes.

Is the study design appropriate for the research question?

Yes

Is the rationale for, and objectives of, the study clearly described?

Partly

Are sufficient details of the methods provided to allow replication by others?

Partly

Are the datasets clearly presented in a useable and accessible format?

Partly

Reviewer Expertise:

Oral health and general health, oral health promotion, dental public health

I confirm that I have read this submission and believe that I have an appropriate level of expertise to confirm that it is of an acceptable scientific standard, however I have significant reservations, as outlined above.
